# Targeting of Herpes Simplex Virus 1 Thymidine Kinase Gene Sequences into the OCT4 Locus of Human Induced Pluripotent Stem Cells

**DOI:** 10.1371/journal.pone.0081131

**Published:** 2013-11-29

**Authors:** Wu Ou, Pingjuan Li, Jakob Reiser

**Affiliations:** Division of Cellular and Gene Therapies, Center for Biologics Evaluation and Research, FDA, Bethesda, Maryland, United States of America; University of Newcastle upon Tyne, United Kingdom

## Abstract

The *in vitro* differentiation of human induced pluripotent stem cells (hiPSC) to generate specific types of cells is inefficient, and the remaining undifferentiated cells may form teratomas. This raises safety concerns for clinical applications of hiPSC-derived cellular products. To improve the safety of hiPSC, we attempted to site-specifically insert a herpes simplex virus 1 thymidine kinase (HSV1-TK) suicide gene at the endogenous OCT4 (POU5F1) locus of hiPSC. Since the endogenous OCT4 promoter is active in undifferentiated cells only, we speculated that the HSV1-TK suicide gene will be transcribed in undifferentiated cells only and that the remaining undifferentiated cells can be depleted by treating them with the prodrug ganciclovir (GCV) prior to transplantation. To insert the HSV1-TK gene at the OCT4 locus, we cotransfected hiPSC with a pair of plasmids encoding an OCT4-specific zinc finger nuclease (ZFN) and a donor plasmid harboring a promoter-less transgene cassette consisting of HSV1-TK and puromycin resistance gene sequences, flanked by OCT4 gene sequences. Puromycin resistant clones were established and characterized regarding their sensitivity to GCV and the site of integration of the HSV1-TK/puromycin resistance gene cassette. Of the nine puromycin-resistant iPSC clones analyzed, three contained the HSV1-TK transgene at the OCT4 locus, but they were not sensitive to GCV. The other six clones were GCV-sensitive, but the TK gene was located at off-target sites. These TK-expressing hiPSC clones remained GCV sensitive for up to 90 days, indicating that TK transgene expression was stable. Possible reasons for our failed attempt to selectively target the OCT4 locus are discussed.

## Introduction

Human induced pluripotent stem cells (hiPSC) and human embryonic stem cells (hESC) have the capacity to proliferate indefinitely and to differentiate into cells of all three germ layers. Thus, they provide promising resources for regenerative medicine and other applications such as disease modeling and drug screening. HiPSCs are attractive because autologous cellular products can be derived from a patient's own iPSC, minimizing the risk of immunogenicity and graft rejection, and because hiPSC can be derived from somatic cells, avoiding the need to destroy embryos. However, recent reports have raised concerns regarding the safety of these cells for clinical applications [1]. For example, the *in vitro* differentiation of hiPSC to generate specific types of cells is inefficient, and the remaining undifferentiated cells may form teratomas [1], [2], [3].

A number of strategies have been explored to decrease the presence of undifferentiated cells and to mitigate the risk of teratoma formation. These strategies include extending the time of *in vitro* differentiation [4], [5], use of positive and/or negative selection markers [6], [7], [8], [9], [10], and suicide genes. The herpes simplex virus 1 thymidine kinase (HSV1-TK) gene is a commonly used suicide gene for ESC [11], [12], [13], [14], [15]. In addition to ESC, the HSV1-TK suicide gene has also been tested in the context of iPSC [15], [16], [17], [18]. Ganciclovir (GCV), the prodrug of HSV1-TK, can be converted to cytotoxic GCV-triphosphate by HSV1-TK, thereby killing HSV1-TK-expressing cells. In some of the studies reported, teratomas still formed after engraftment even though the stem cells had been engineered to express HSV1-TK and the cellular product had been treated with GCV [13], [14]. One possible reason for this outcome is that the TK gene in the engineered cells mutated over time, a frequent observation for HSV1-TK-expressing tumor cells [19], [20], [21], [22]. In addition, promoter silencing may have been an issue [23].

In this study, we attempted to site-specifically insert a HSV1-TK transgene sequence into the OCT4 locus of hiPSC using an OCT4-specific ZFN pair (OCT4 ZFN#2) that was previously described by Hockemeyer et al. [24]. The OCT4 locus was chosen for three main reasons. 1) The endogenous OCT4 promoter is less likely to be silenced compared to an exogenously added promoter. 2) The OCT4 protein is a core pluripotency factor which is expressed only in undifferentiated stem cells, teratomas and some tumors [25], [26], [27], [28], [29], [30], [31], [32]. Therefore, expression of HSV1-TK from the endogenous OCT4 promoter, followed by GCV treatment is expected to help ablate not only teratomas but also other tumors that may develop after transplantation. 3) A study reported before by Hockemeyer et al. has shown that promoter-less GFP transgene constructs can be successfully knocked into the first intron of the OCT4 gene using a zinc finger nuclease approach [24]. Expression of GFP fused to sequences encoded by the first exon of OCT4 did not affect the stem cell's pluripotency [24]. HSV1-TK was chosen primarily because of its track record in stem cell research and because it was shown to be immunogenic [33]. An immune response to the HSV1-TK expressing cells would be a desirable outcome since it would provide an additional layer of safety. Here we present results documenting site-specific insertion of a HSV1-TK/puromycin resistance gene cassette at the OCT4 locus in hiPSC. However, none of the correctly targeted hiPSC clones displayed TK activity, judged by their sensitivity toward GCV.

## Materials and Methods

### Chemicals

The Y-27632 ROCK inhibitor was obtained from CalBioChem/Millipore (Billerica, MA) or Sigma-Aldrich (St. Louis, MO). A 10 mM stock solution was prepared by dissolving 1 mg of Y-27632 in 299 µl of DMSO. Aliquots (50 µl) were stored at −20°C. Thiazovivin was obtained from Stemgent (Cambridge, MA). A 10 mM stock solution was prepared by dissolving 1 mg of thiazovivin in 321 µl of DMSO. Aliquots (50 µl) were stored at −20°C. Ganciclovir was obtained from Sigma-Aldrich. A 40 mM stock solution was prepared by dissolving 100 mg of GCV in 9.8 ml of 0.1 M HCl. The solution was stored at 4°C.

### Cell culture

**Figure 1:**
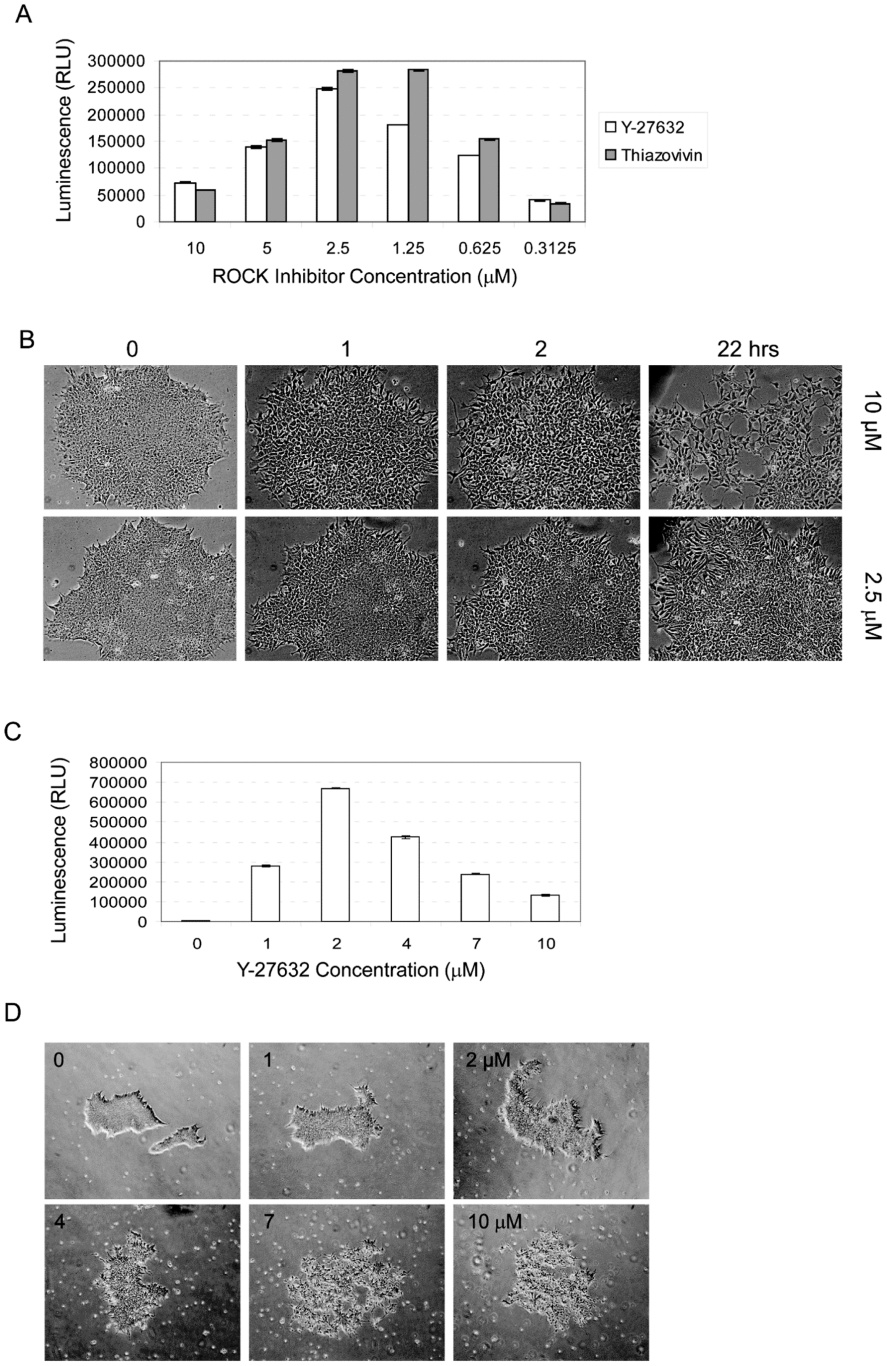
Optimization of ROCK inhibitor concentrations. A. Viability of DF19 iPSC four days after plating following pre-treatment for one hour at the indicated concentrations of the ROCK inhibitors Y-27632 or thiazovivin. B. Morphology of DF19 iPSC treated using 2.5 (top panel) or 10 µM (bottom panel) of the ROCK inhibitor Y-27632 for various lengths of time. Pictures were taken at the indicated treatment time points. C. Viability of ND2.0 iPSC four days after plating following pre-treatment for one hour at the indicated concentrations of the thiazovivin inhibitor. D. Morphology of ND2.0 iPSC treated using increasing concentrations of the thiazovivin inhibitor.

The human DF19-9-11T.H iPS cell line [34], referred to as DF19 for simplicity, was obtained from the WiCell Research Institute (Madison, WI). The ND2.0 hiPSC line produced in Dr. James Thomson's laboratory at the University of Wisconsin was a kind gift from Dr. Guokai Chen (NHLBI/NIH, Bethesda, MD) (http://www.crm.nih.gov/researchTools/availableCels.asp, http://www.nature.com/nmeth/journal/v8/n5/full/nmeth.1593.html?WT.ec_id=NMETH-201105). The BC1 hiPSC line [35] was obtained from the NIH Center for Regenerative Medicine (CRM), Bethesda, MD. Cells were maintained in mTeSR1 medium (StemCell Technologies, Vancouver, Canada) in NUNC six well plates (Fisher Scientific, Hanover Park, IL), coated with Matrigel (BD Biosciences, San Jose, CA), with daily medium changes. The cells were passaged using Accutase (Millipore, Billerica, MA), Dispase (Invitrogen, Grand Island, NY), or 0.5 mM EDTA [36]. For passage as single cells or small clumps, cells were pretreated with the ROCK inhibitors for 1 hour at the concentrations indicated in Fig. 1. The ROCK inhibitors were also added to the medium for the first 24 hours after passage. To treat cells with GCV, the stock solution was diluted with mTeSR1 medium to 2 µM before adding to the cells.

### Plasmids

The OCT4-eGFP-2A-Puro donor plasmid (Plasmid # 31939; Addgene, Cambridge, MA) [24] was kindly provided by Dr. Rudolf Jaenisch, the Whitehead Institute for Biomedical Research, Cambridge, MA. The OCT4-TK-2A-Puro donor plasmid was constructed as follows: A NheI site was first introduced in the parental OCT4-eGFP-2A-Puro donor plasmid at the beginning of the eGFP coding region using site-directed mutagenesis and the primers listed in Table 1. The HSV1-TK SR39 sequence [37] was codon optimized for expression in human cells and synthesized by GeneArt (Invitrogen), and sub-cloned into the donor plasmid between the NheI and SalI restriction sites. The final plasmid was confirmed by sequencing. The coding regions for the OCT4-specific ZFN pair (OCT4 ZFN#2) was codon optimized for expression in human cells and synthesized by DNA2.0 (Menlo Park, CA), according to the published amino acid sequences [24]. The synthetic DNA fragments were subcloned between the AgeI and XhoI restriction sites present in the mammalian expression plasmid pJ603 (DNA2.0).

**Table 1:** Primers used for mutagenesis and for amplifying the 5′ and 3′ junction sequences, TK coding sequence, and plasmid backbone sequences.

Primer Name	Sequences (5′ to 3′)
NheI-F	GAACAGCTCCTCGCCCTTGCTAGCCATGGCCTGTGGGAGGAAG
NheI-R	CTTCCTCCCACAGGCCATGGCTAGCAAGGGCGAGGAGCTGTTC
p1	TTATCGGGGAAGAAGTGGCTG
p2	AGCCGATTGTCTGTTGTGCC
p3	TCCTCGTGCTTTACGGTATCG
p4	CACGATGCCTGTAGCAATGG
p5	TTGTTGTGGCCTCTGCTTCTG
p6	ATCTTCTGTTCCAGCCGCACT
p7	CAAATGTGTCAGTTTCATAGCCTG
p8	AGCAGCCTCTGTTCCACATACACT
p9	GAAAGTGCACTTGGAAGAGATCC
p10	CAGGGCTCTTTGTCCACTTTGTA
p11	CTTATCCCAGCAGAACTGAGGAAT
p12	AGCTTTGGACTTGCTGAGTAACAG
p13	GCCCAGCAAAGAACTTCTAACCTT
p14	CATAACCTGACAGGTGTTCTCCTC
p15	ACGTCTCCCGCAAGCTTAAG

The relative locations of these primers are shown in Fig. 4A

### Nucleofection of hiPS cells and derivation of puromycin-resistant cell clones

The human iPS cell lines DF19, ND2.0 and BC1 were pretreated with the ROCK inhibitor Y-27632 (2 µM) for one hour and dissociated with 5 µM EDTA to generate single cells. For nucleofection, 1×10^6^ cells were aliquoted into 15-ml Falcon tubes and pelleted by centrifugation at 200×*g* for 5 minutes. Cell pellets were resuspended in 100 µl of Solution I or II (Human Stem Cell Nucleofection Kit 1 and 2) (Lonza, Allendale, NJ). For nucleofection, various amounts of the two OCT4 ZFN#2 plasmids (0.25–2 µg each), donor plasmids (2.5–10 µg each), or control pmaxGFP plasmid (2 µg, Lonza) were added to Solution I or II first. Nucleofection was performed using the A012, A013, A023, A027 or B016 programs of the Nucleofector 2b Device (Lonza). Cells from each nucleofection were immediately resuspended in 3 ml of pre-warmed mTeSR1 medium supplemented with 2 µM ROCK inhibitor Y-27632, plated and cultured in 6-well plates. After 24 hours, the medium was replaced with regular mTeSR1 medium. Two days after nucleofection, cells were harvested and resuspended in culture medium and divided into two equal aliquots. One of the cell aliquots was analyzed by FACS to determine the percentage of GFP positive cells; and the other aliquot was analyzed for cell viability using the CellTiter-Glo luminescence assay kit (Promega, Madison, WI). Use of Solution I and program B016 resulted in a high percentage of GFP positive cells and high cell viability. All subsequent studies were conducted using these conditions. Two to four days after nucleofection, cells were selected with 0.1–0.3 µg/ml puromycin for 3–5 days. The surviving clones were manually picked and expanded in mTeSR1 medium, which was supplemented with 2 µM ROCK inhibitor Y-27632 during the first 24 hours.

### PCR analysis of hiPSC clones

Genomic DNA derived from individual hiPSC clones or the parental hiPSC cell lines was extracted using the Qiagen DNeasy Blood & Tissue Kit (Qiagen, Valencia, CA) according to the product manual. Genomic DNA of human newborn foreskin fibroblasts (ATCC Manassas, VA; Cat.# CRL-2097) was kindly provided by Dr. Rachel Goehe (FDA/CBER). The insertion junction sequences, TK coding sequences and donor plasmid backbone sequences were PCR amplified using Platinum Taq DNA polymerase (Invitrogen) and subjected to DNA sequencing. The following conditions were used for PCR: 94°C, for 2 min; followed by 35 cycles consisting of 94°C, 30 sec, 55°C, 30 sec, 72°C, 90 sec; followed by a 10-min incubation at 72°C. The primers used are listed in Table 1.

### Western blot analysis

Cells were lysed in RIPA buffer supplemented with the Complete Mini Protease Inhibitor Cocktail (Roche Applied Science, Indianapolis, IN). The proteins were separated on a pre-cast NuPAGE 4–12% Bis-Tris gel and transferred to PVDF membranes (Invitrogen). The primary antibodies used included a mouse anti-OCT4 monoclonal antibody (Cat. # sc-5279, Santa Cruz Biotechnologies, Dallas, TX), prediluted 1∶500, or goat anti-TK antibody (Cat. # sc-28038, Santa Cruz Biotechnologies) prediluted 1∶1000. The anti-OCT4 monoclonal antibody was raised against amino acids 1–134 of human OCT4, corresponding to the first exon of the OCT4 gene. The secondary antibodies used were HRP-conjugated anti-mouse IgG and anti-goat IgG (Invitrogen), both prediluted 1∶5000.

## Results

### Optimization of ROCK inhibitor concentration

Rho kinase (ROCK) inhibitors are widely used to improve the viability of stem cells, especially when they are passaged as single cells or small clumps and during freeze/thaw [38], [39], [40]. Typically, ROCK inhibitors are used at a concentration of 10 µM [38], [39], [40]. Since the optimal ROCK inhibitor concentration may vary for different hiPSC cell lines, the optimal concentration of the ROCK inhibitor Y-27632 for the DF19 iPSC line was determined. To do this, cells were pretreated with the Y-27632 inhibitor supplied by CalBioChem/Millipore for 1 hour at concentrations ranging from 0.3125 µM to 10 µM. The cells were then transferred as single cells/small clumps in medium that contained the same concentration of the Y-27632 inhibitor for 24 hours. Cell viability was determined four days post passage using the CellTiter-Glo luminescence viability assay. This assay determines the number of viable cells in culture based on quantitation of the ATP present, which signals the presence of metabolically active cells. The number of viable cells was highest when the Y-27632 inhibitor was used at a concentration of 2.5 µM (Fig. 1A). The same effect was seen using the Y-27632 inhibitor provided by Sigma-Aldrich (data not shown) or thiazovivin, a different ROCK inhibitor (Fig. 1A). To determine the effects of the Y-27632 inhibitor on cell morphology, DF19 cells were treated with Y-27632 for various lengths of time two days after plating. Consistent with the viability data presented in Fig. 1A, the colony morphology changed dramatically over time using the Y-27632 inhibitor at a concentration of 10 µM. These changes started to emerge within one hour (Fig. 1B, top row). For cells exposed to 2.5 µM Y-27632, the cell morphology changes were much less pronounced (Fig. 1B, bottom row). Similar results were found with the hiPSC cell line ND2.0. Cell viability was highest when 2 µM of the Y-27632 was used (Fig. 1C), and changes in colony morphology were apparent at concentrations above 2 µM (Fig. 1D). Based on these findings, Y-27632 at a concentration of 2 µM was chosen for the rest of this study.

### Optimization of nucleofection conditions for the DF19 hiPSC line

**Figure 2:**
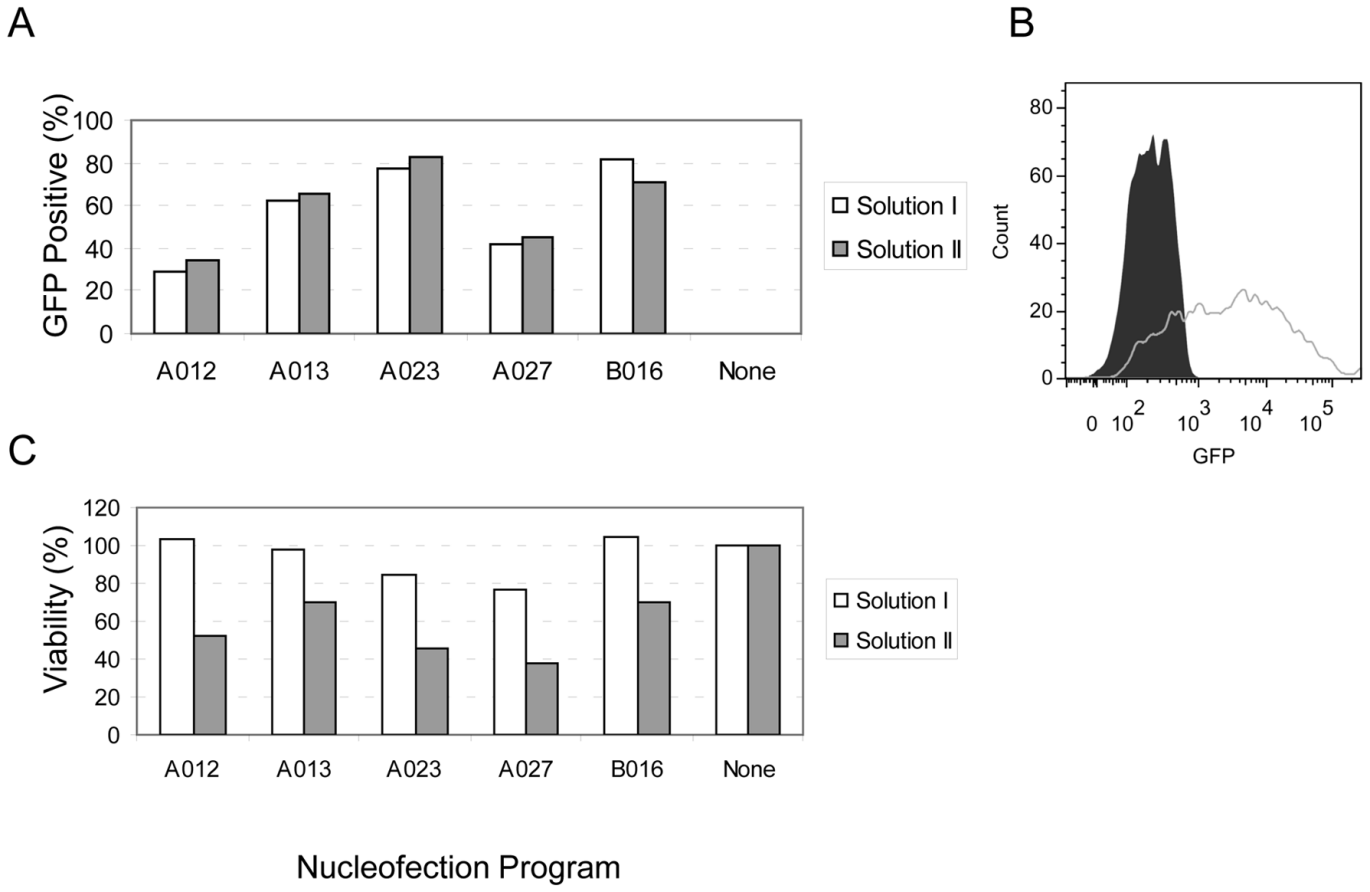
Optimization of nucleofection conditions. A. Percentage of GFP-positive cells. One million DF19 cells were transfected with 2 µg of the pmaxGFP plasmid using the Nucleofector 2b device and different Nucleofection solutions and programs. Two days post transfection, the percentage of GFP positive cells was determined using FACS. B. FACS profile of cells nucleofected using Solution I and program B016. The gray line represents transfected cells and the black graph untransfected cells. C. Cell viability. Cell viability was determined using Promega's CellTiter-Glo kit. The viability of control cells that were not nucleofected but otherwise treated in the same way was taken as 100%.

To optimize the nucleofection conditions for the DF19 iPSC line, the pmaxGFP plasmid and two commercial Nucleofection kits and different nucleofection programs were used. Solution I and program B016 resulted in the highest percentage of GFP positive cells (Fig. 2A), and the cells appeared bright by FACS (Fig. 2B), while programs A012 or B016 and Solution I resulted in a higher cell viability (Fig. 2C). Taking into account both the transfection efficiency and cell viability, program B016 and Solution I were chosen for the rest of this study.

### Test of ZFN targeting strategy in DF19 iPSC

**Figure 3:**
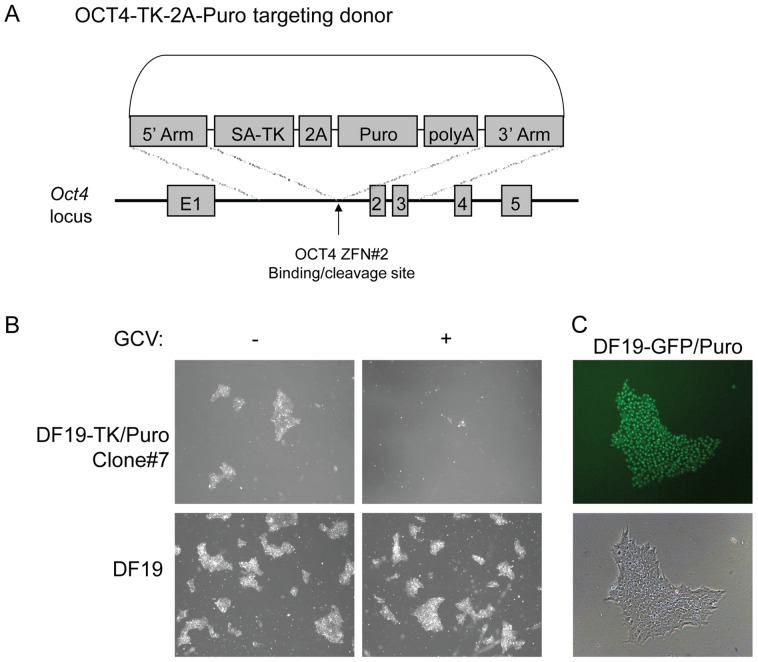
Test of ZFN targeting strategy in DF19 cells. A. Overview depicting the targeting strategy for the OCT4 locus. B. Panels displaying one of the puromycin-resistant cell clones (DF19-TK/Puro Clone #7) and unmodified DF19 cells after treatment with or without 2 µM GCV for 2 days. Bright field pictures were taken using a 2× objective. C. Panels showing one of the clones (DF19-GFP/Puro) obtained using the OCT4-eGFP-2A-Puro control plasmid.

In an attempt to target HSV1-TK transgene sequences to the OCT4 locus, DF19 iPSC were nucleofected using plasmids encoding the OCT4 ZFN#2 and the OCT4-TK-2A-Puro donor targeting plasmid. In parallel, plasmids encoding the OCT4 ZFN#2 and the OCT4-eGFP-2A-Puro donor targeting plasmid [24] were used. In these targeting constructs, the TK-2A-Puro and GFP-2A-Puro transgene sequences are preceded by a splice acceptor (SA) sequence to mediate splicing of the TK-2A-Puro and GFP-2A-Puro transgene sequences to the first exon of the endogenous OCT4 gene (Fig. 3A). This will result in fusion proteins consisting of a protein domain encoded by the first exon of the OCT4 gene linked to TK and GFP sequences, respectively, expressed from the endogenous OCT4 promoter. Stable transfectants were selected using puromycin. Forty four puromycin-resistant clones were obtained for the OCT4-TK-2A-Puro donor plasmid. Four of these clones (#2, 3, 7 and 8) were found to be sensitive to GCV. One representative clone (#7) is shown in Fig. 3B. Nine puromycin-resistant clones were obtained for the OCT4-eGFP-2A-Puro control plasmid, and one of them was GFP-positive (Fig. 3C).

### Characterization of puromycin resistant DF19 iPSC clones

In previous studies, TK-expressing stem cells were found to form teratomas even after treating them with GCV [13], [14]. One possible reason may be that the TK-expressing stem cells developed resistance to GCV as what was described before for TK-expressing tumor cells [19], [20], [21], [22]. To determine whether DF19-TK-2A-Puro cells have the capacity to develop resistance to GCV, clones #2, 7 and 8 were periodically tested for GCV resistance over a period of 90 days. No GCV-resistant cells were found (data not shown), suggesting that the cells' sensitivity toward GCV did not change.

**Figure 4:**
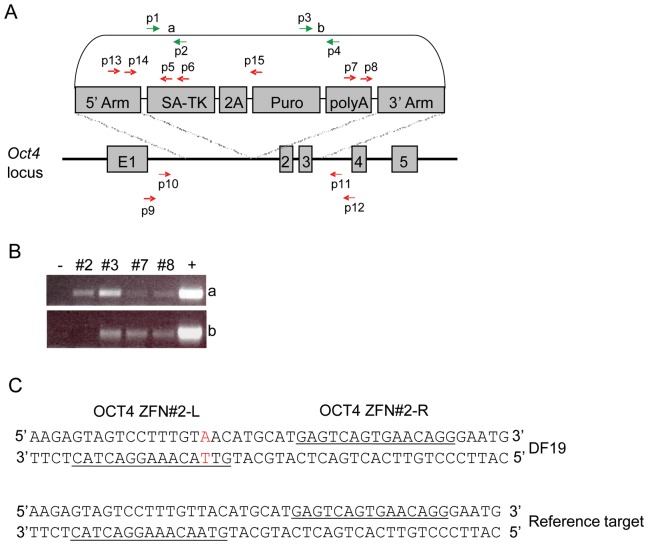
PCR analysis of DF19 iPSC clones. A. Schematic overview depicting the OCT4-TK-2A-Puro donor plasmid and PCR primers used. B. PCR analysis of genomic DNA from DF19 clones #2, 3, 7 and 8 using the p1/p2 and p3/p4 primer pairs specific for the plasmid backbone. The resulting PCR products are referred to as “a” and “b”. C. Analysis of the target sequence for the OCT4 ZFN#2. The binding sites for the left (L) and right (R) ZFN are underlined. The single-nucleotide change in DF19 genomic DNA is shown in red.

We next wanted to determine whether the TK-2A-Puro cassette in clones #2, 3, 7, and 8 was targeted correctly to the OCT4 locus. To do this, we performed a series of nested PCR analyses using primer sets that cover the putative 5′ and 3′ junction sequences (Fig. 4A and Table 1). Primer sets p9/p6 and p10/p5 were used to amplify the 5′ junction sequence in clones #2, 3, 7, and 8, and primer sets p7/p12 and p8/p11 for the 3′ junction sequence. There were no detectable PCR products for any of the four TK-2A-Puro clones or the GFP-2A-Puro clone tested (data not shown). However, use of the p1/p2 and p3/p4 primer pairs that are specific for sequences present in the backbone of the donor plasmid (referred to as sequences “a” and “b”, respectively) (Fig. 4A) resulted in the expected PCR products for all of four TK-2A-Puro clones except for clone #2 that did not produce a PCR product using the p3/p4 primer set (Fig. 4B). Taken together, the PCR results suggested that the entire donor plasmid had integrated at sites other than the OCT4 locus for all the four clones tested. These findings were confirmed by Southern blotting using a digoxigenin-labeled TK probe and genomic DNA extracted from clones #2, 3, 7, and 8. The results obtained indicated that they all contained at least two copies of the TK gene sequence, each at different locations (data not shown).

A Western blot analysis involving cell extracts prepared from clones #2, 3, 7, and 8 and the parental cells showed that OCT4 levels in the four cell clones and in the parental cells were similar. In agreement with the PCR results, there was no evidence for a fusion protein encoded by the first exon of the OCT4 gene and the TK transgene following probing of the Western blots using anti-OCT4 or anti-TK antibody (data not shown).

To investigate possible reason(s) for our failure to target the OCT4 locus, we analyzed the sequence of the OCT4 ZFN#2 target site sequence present in DF19 cells. Compared to the sequence of the OCT4 ZFN#2 target site present in the human BG01 ESC line that was used by Hockemeyer et al. [24], a single-nucleotide change was found in the left ZFN binding site present in the DF19 iPSC line (Fig. 4C). The same single-nucleotide change was found in human newborn foreskin fibroblasts (ATCC, CRL-2097) that were originally used to generate the DF19 iPSC line [34] (data not shown). It remains to be determined whether this single base pair change in the left ZFN binding site may have contributed to the ZFN's lack of targeting specificity.

### Targeting of TK sequences to the OCT4 locus in BC1 iPSC

**Figure 5:**
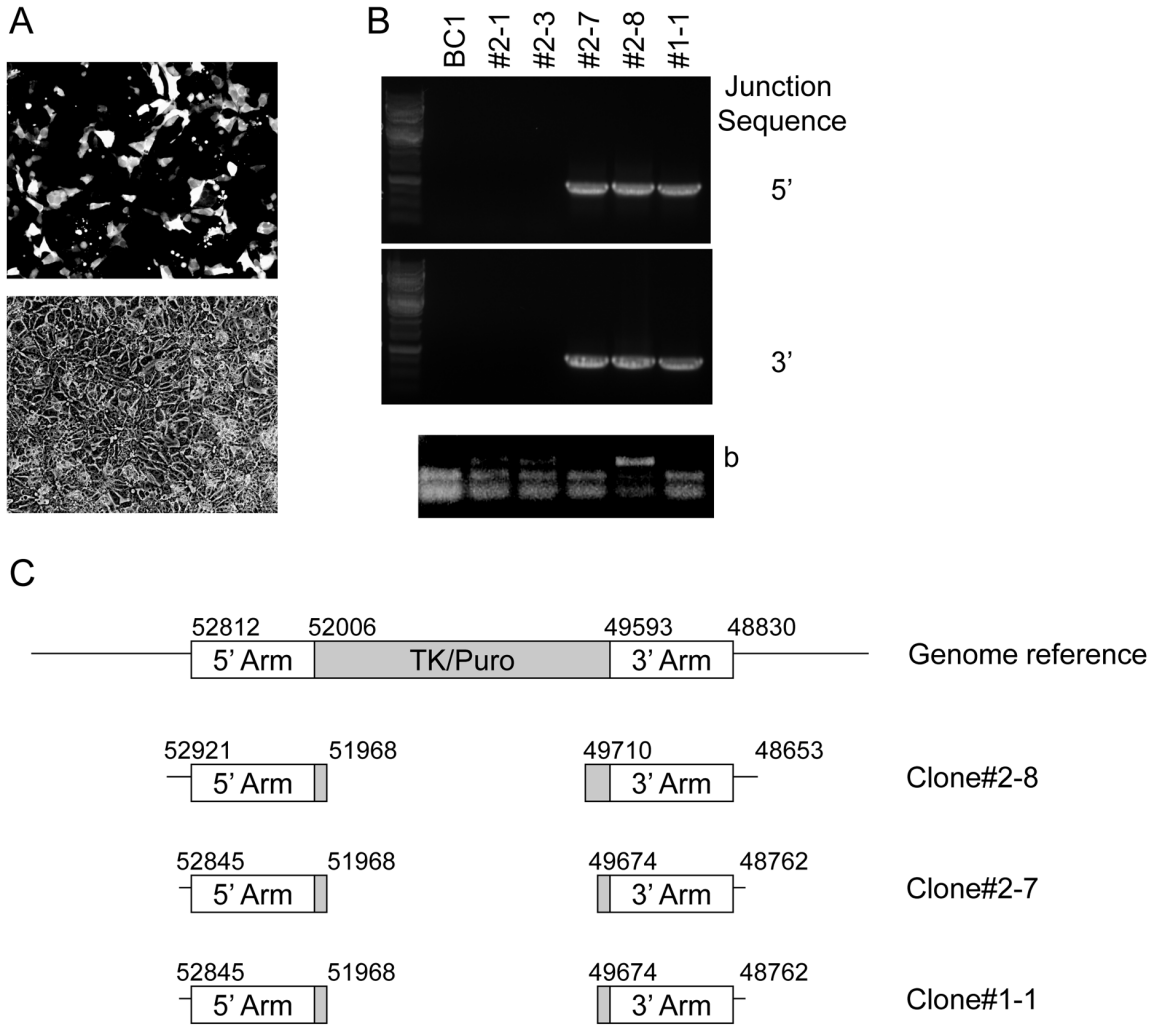
Analysis BC1 iPSC clones. A. Nucleofection of BC1 iPSC using the pmaxGFP plasmid. Top panel: Fluorescence image of transfected cells shown in grayscale; Bottom panel: Bright field image. B. Nested PCR amplification of genomic insertion sites (top and middle panels) and donor plasmid backbone sequence (bottom panel) using primers shown in Fig. 4A. Primers p9/p6 and p10/p5 were used to amplify the 5′ junction sequence and primers p7/p12 and p8/p11 for the 3′ junction sequence. Each clone was passaged at least 6 times before genomic DNA was isolated. C. Analysis of insertion junction sequences for clones #2-8, 2-7 and 1-1. The numbers above the diagrams reflect arbitrary numbers. The 5′ and 3′ homology arms extending from nucleotides 52006 to 52812 and nucleotides 48830 to 49593, respectively are depicted as open boxes. TK/Puro sequences are highlighted in gray. The PCR products for clones # 2-8, 2-7, and 1-1 spanning the 5′ or 3′ arms plus flanking sequences were-subcloned and sequenced. The numbers indicate the regions that were confirmed by sequencing.

The failure to obtain DF19 cell clones with the TK-2A-Puro transgene correctly inserted at the OCT4 locus may have been caused in part by the sequence mismatch affecting the OCT4 ZFN#2 binding. Therefore, other iPSC were screened in an attempt to identify cell lines with the correct target sequence. The BC1 cell line was confirmed by DNA sequencing to contain the correct OCT4 ZFN#2 binding site (data not shown). It was then subjected to nucleofection to determine whether puromycin-resistant cell clones with the TK-2A-Puro transgene cassette correctly inserted at the OCT4 locus could be obtained. As shown in Fig. 5A, the nucleofection efficiency using the pmaxGFP plasmid was high for BC1 cells. After nucleofection using the TK-2A-Puro donor plasmid and plasmids encoding the OCT4 ZFN#2, 10 puromycin-resistant cell clones were obtained. There were no colonies when the cells were transfected with the donor plasmid alone. Five of these clones were lost during expansion because of spontaneous differentiation or because they grew slowly. Of the five remaining clones (clones #1-1, 2-1, 2-3, 2-7 and 2-8), two (clones #2-1 and 2-3) were found to be sensitive to GCV. To analyze the fate of TK-2A-Puro donor plasmid-derived sequences in the puromycin-resistant cell clones, we performed a series of nested PCR reactions using primers specific for the junction sequences or sequences corresponding to the backbone of the donor plasmid as outlined in Fig. 4A. Primers p9/p6 and p10/p5 were used to amplify the 5′ junction sequence and primers p7/p12 and p8/p11 for the 3′ junction sequence. The PCR reactions for the junction sequences were negative for clones #2-1 and 2-3 but positive for clones #1-1, 2-7 and 2-8 (Fig. 5B). The PCR reactions to detect the backbone sequences of the donor plasmid were positive for clones #2-1, 2-3 and 2-8, and negative for clones #1-1 and 2-7. The results obtained suggest that, based on PCR, the TK-2A-Puro sequences in clones #1-1 and 2-7 were correctly targeted to the OCT4 locus but not in clones #2-1 and 2-3. For clone #2-8, there was one TK-Puro sequence at the OCT4 locus and an additional sequence containing the plasmid backbone at an off-target site. To confirm that clones #1-1, 2-7 and 2-8 contained correctly targeted TK-Puro sequences, the PCR products for the junction sequences were cloned into the pCR2.1-TOPO vector and sequenced. As referred to in Fig. 5C, all sequences revealed the predicted junctions.

Surprisingly, all of the correctly targeted clones (i.e. clones #1-1, 2-7 and 2-8) survived exposure to 2 µM GCV, indicating that TK expression in these clones was either low or absent, possibly caused by changes in the TK transgene sequence, while the two off-targeted clones (clones #2-1 and 2-3) were GCV-sensitive, indicating that the TK transgene was expressed in these clones. To investigate whether increased concentrations of GCV would result in the killing of clones #1-1, 2-7 and 2-8, GCV at a concentration of 4 µM was tested. GCV at this concentration was found to nonspecifically inhibit the growth of both the engineered cells and the parental cells but did not lead to the selective killing of cells derived from clones #1-1, 2-7 and 2-8 (data not shown).

In addition to the junction sequences (Fig. 5C), the full-length TK-encoding transgene sequences in the targeted clones was also confirmed by DNA sequencing following nested PCR amplification using primers p13/p15 and p14/p15 (Fig. 4A,). The characteristics of all the BC1 TK-2A-Puro clones analyzed are summarized in Table 2.

**Table 2:** Characteristics of BC1 cell-derived clones.

	BC1	#2-1	#2-3	#2-7	#2-8	#1-1
GCV Sensitivity	−	+	+	−	−	−
Sequence TK gene	NA	ND	ND	+	+	+
PCR 5′ Junction Sequence	NA	−	−	+	+	+
PCR 3′ Junction Sequence	NA	−	−	+	+	+
PCR Donor Backbone	NA	+	+	−	+	−

NA: Not applicable

ND: Not done

+: Sequence verified by PCR

-: PCR did not reveal the expected fragment

## Discussion

Homologous recombination (HR) approaches involving engineered nucleases have emerged as powerful tools for site-specific insertion of transgene sequences in the genomes of mammalian cells [41]. The aim of the work reported here was to use a HR approach to insert a transgene cassette encoding HSV1-TK fused to a puromycin resistance protein into intron 1 of the OCT4 gene, using a ZFN pair that was used before to insert GFP transgene sequences at the OCT4 locus in human ESC [24]. A HR strategy to knock in the TK transgene into the OCT4 locus of mouse ESC was described before by Hara et al. [13]. GCV-mediated killing of ESC indicated that the TK transgene was expressed in such cells at sufficiently high levels to mediate cell killing [13]; however, the transgene insertion site was not analyzed. Our results obtained using two different iPSC lines indicated that very few puromycin-resistant cell clones were obtained with HSV1-TK transgene sequences inserted correctly at the OCT4 locus and that none of these cell clones were sensitive to GCV. This is consistent with the view that the TK transgene was not expressed in these clones, or at low levels only. A DNA sequence analysis of the HSV-1 TK transgene sequence in these clones revealed that the sequence was correct. This shows that the lack of TK transgene expression in these clones was not caused by changes in the TK transgene sequence. Interestingly, all the clones containing off-targeted HSV1-TK transgene sequences were GCV sensitive, indicating that the HSV1-TK transgene was expressed under these conditions.

A number of factors may have contributed to the low targeting efficiency. For example, the OCT4 ZFN#2 binding site in DF19 cells was found to differ from the original target sequence reported by Hockemeyer et al. [24] by a single nucleotide change. This may have impacted the performance of the ZFN in DF19 cells. This underlines the importance to verify the target sequence in a particular cell line early on when ZFN approaches are being pursued. Also, it is conceivable that expression of HSV1-TK as a fusion protein involving sequences encoded by the first exon of the OCT4 gene may have impacted its enzymatic activity. Alternatively, it is conceivable that TK transgene integration may have negatively impacted transcription from the OCT4 promoter, perhaps by inadvertently removing a cis-regulatory sequence within the OCT4 locus itself.

There are a number of options to improve the targeting efficiency of suicide gene sequences to put them under the control of a stem cell-specific or constitutive, endogenous promoter. 1) Use of ZFNs that target alternative genomic sites. As shown in the study reported by Hockemeyer et al. [24], GFP transgene expression levels varied greatly depending on the site of insertion of the GFP transgene cassette in the vicinity of the OCT4 gene. 2) Other genome editing tools such as TALENs [42] may be considered to place HSV1-TK transgene sequences downstream of a stem cell-specific or a constitutive promoter [24]. Use of endogenous, stem cell-specific promoters to drive suicide gene expression may not always be advantageous, since certain endogenous promoters (such as the OCT4 promoter) may also be active at low levels in progenitors of certain lineages [43], [44]. These progenitor cells may, in some cases, be important for successful engraftment and their loss following exposure to the prodrug may jeopardize engraftment. To avoid this from happening, one would need to first carefully examine whether the promoter is active in the progenitor cells of interest. In light of this potential drawback, constitutive promoters involving genomic “safe harbor” sites such as AAVS1 locus [45], [46] should be explored as a backup. 3) Besides HSV1-TK, other suicide genes like inducible caspase 9 [47] may be worth pursuing.

Mutations affecting TK function are frequently observed in tumor cells [19], [20], [21], [22]. Encouragingly, none of the three DF19-TK-2A-Puro clones (#2, 7, and 8) tested developed GCV resistance upon exposure to GCV for up to 90 days. It remains to be tested, though, whether the expression of TK from a correctly targeted locus of interest will be equally stable.
